# Hyponatremia due to excessive water intake in COVID-19 patients: case series study

**DOI:** 10.1186/s43162-022-00158-0

**Published:** 2022-09-23

**Authors:** Ahmad Nasrat Al-juboori, Amira Al Hail, Zaid Ahmad Al-juboori

**Affiliations:** 1 Otorhinolaryngology, Head & Neck Surgery (ORL-HNS) Department, Al Wakra Hospital, Al Wakra, Qatar; 2 Hamad Medical Corporation, P.O. Box 3050, Doha, Qatar; 3 Weill Cornell Medical College, Ar-Rayyan, Qatar; 4 College of Medicine, Qatar University, Doha, Qatar

**Keywords:** Hyponatremia, SARS-CoV-2, COVID-19, Excessive water intake, Dysgeusia

## Abstract

**Background:**

Literatures revealed syndrome of inappropriate antidiuretic hormone (SIADH) as the most common mechanism of hyponatremia in COVID-19. However, it is important to rule out other etiologies of hyponatremia.

**Methods:**

This is a case series, retrospective study. Four patients were reviewed from the Mesaieed Hospital, Hamad Medical Corporation, Qatar. The patients were admitted during the second wave of pandemic because of mild to moderate COVID pneumonia. The patients received medications according to the protocol; after few days of treatment, their blood laboratory results showed hyponatremia; as compared to the initial readings, hyponatremia workup excluded SIADH. History revealed that the patients were drinking large amounts of water, around 4–5 L/day, due of certain reasons: one patient had dysgeusia, and the other three patients thought that excessive drinking of water is beneficial for COVID-19 infection.

**Results:**

The hyponatremia level was less than 135 mmol/L, other laboratory tests excluded SIADH, and the provisional diagnosis was dilutional hyponatremia. Male/female ratio was 3/1, age from 29- to 45-year-old patients with no associated comorbidities. Fluid restriction up to 1.5 L/day showed dramatic improvement of their sodium blood level. The patients are discharged in a stable condition.

**Conclusions:**

In COVID-19 patients, hyponatremia not only is secondary to SIADH but can also be due to other etiologies. Hyponatremia can be induced by excessive water drinking and considered an extremely rare reported cases.

## Background

**Fig. 1:**
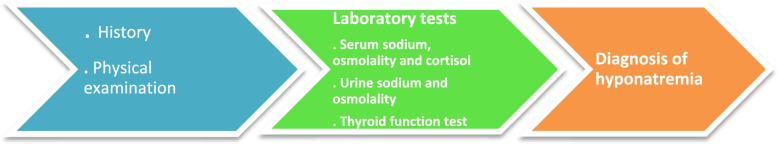
Diagnosis of hyponatremia

Hyponatremia is one of the most frequently observed electrolyte disturbances in coronavirus disease 2019 (COVID-19). Literature describes syndrome of inappropriate antidiuretic hormone (SIADH) as the mechanism of hyponatremia in COVID-19 requiring fluid restriction for management. However, it is important to rule out other cause of hyponatremia in such cases keeping in consideration the effect of an alternate etiology on patient management and outcome [1, 2]. Hyponatremia is defined when serum sodium level less than 135 mmol/L. In many cases, this electrolytic abnormality is caused by a variety of factors [3, 4]. Hyponatremia is the most common electrolyte disorder seen in clinical practice and is associated with increased risk of morbidity and mortality [5, 6]. This electrolytic disequilibrium is classified in hypovolemic, euvolemic, and hypervolemic hyponatremia, each category’s therapeutic approach being different [7]. The most recognizable cause of hyponatremia in COVID-19 is the SIADH; it is found in about 40–50% of patients with this electrolyte disorder [8, 9]. These percentages may be higher in some conditions, such as pneumonia, traumatic head injury, or subarachnoid hemorrhage [10]. In order to establish the diagnosis and etiology of hyponatremia, a careful history and thorough physical examination are required; investigations such as serum sodium level, urine sodium level, serum osmolality, urine osmolality, thyroid function tests, and serum cortisol may be indicated (Fig. 1) [11, 12].

The aim of our study was highlighted on rare cause of hyponatremia happened in COVID-19 patients, which was the dilutional hyponatremia; the reasons for dilutional hyponatremia were due to excessive water intake which is considered as extremely rare reported cases.

## Methods

This is a case series, retrospective study. Four patients were reviewed from the Mesaieed Hospital, which is one of the COVID-19 facilities of the Hamad Medical Corporation, Qatar. The patients were admitted during the second wave of pandemic (from March until July 2021) because of mild to moderate COVID pneumonia. The patients experienced cough and fever, and one of them had shortness of breath (PO2 94%) and one with hyposmia and dysgeusia, dysgeusia manifested by the bitterness sensation of tongue. Vital signs were within normal limits except for fever and low oxygen saturation for one patient. Physical examination revealed a well-hydrated patients, a fully conscious, and oriented, with no focal neurological deficit. The rest of the physical examination was unremarkable except for the patient with dyspnea; there was chest crackle mainly on the basal area. Initial laboratory results are shown in Table 1, with a mild to moderate increase in C-reactive protein. Chest X-ray showed a typical ground-glass appearance mainly on the right side for three patients. They received medication according to the protocol; after few days of treatment, their blood laboratory results showed hyponatremia; as compared to the initial readings, hyponatremia workup excluded SIADH. History revealed that the patients were drinking large amounts of water, around 4–5 L/day, because of certain reasons: one patient had dysgeusia, and the other three patients thought that excessive drinking of water is beneficial for COVID-19.

**Table 1:** Laboratory investigations for COVID-19 patients with hyponatremia

Investigation	Patient 1	Patient 2	Patient 3	Patient 4	Normal range
White blood cell count	7.8	10.2	11.1	8.5	4–10 × 10^3/uL
Hemoglobin	17.0	12.3	14.3	15.5	13–17 g/dL
Hematocrit	43.3	44.2	45.1	43.9	40–50%
Lymphocyte count	0.8	1.0	1.3	0.9	1–3 × 10^3/uL
Platelets	152	145	160	155	150–400 × 10^3/uL
Creatinine	65	60	55	61	62–106 umol/L
Alanine aminotransferase	79	50	35	55	0–41 U/L
C-reactive protein	52.8	30.5	25.6	51.3	0–5 mg/L
Procalcitonin	0.18	0.23	0.11	0.44	< 0.5 ng/mL
Lactic acid	2.3	0.3	0.6	1.9	0.5–2.2 mmol/L
PCR COVID-19	Positive	Positive	Positive	Positive	Not applicable
Serum sodium	129	130	131	128	136–145 mmol/L
Serum osmolality	225	220	219	235	275–295 mmol/kg
Urine sodium	36	25	30	32	25–40 mEq/L
Urine osmolality	85	91	101	88	150–1150 mmol/kg
TSH	0.85	1.5	0.99	1.8	0.3–4 mIU/L
Serum cortisol level (AM)	357	299	312	405	133–537 mmol/L

## Results

The hyponatremia level was less than 135 mmol/L, other laboratory tests excluded SIADH, and the provisional diagnosis was dilutional hyponatremia. Male/female ratio was 3/1, age from 29- to 45-year old patients with no associated comorbidities. Fluid restriction up to 1.5 L/day showed dramatic improvement of their sodium blood level. The patients discharged in a stable condition. The studied four-patient blood laboratory results are shown in Table 1.

## Discussion

Hyponatremia is one of the most common electrolyte disorders, which has a high prevalence of morbidity and mortality rates [13], especially in patients suffering from COVID-19 pneumonia. The physiological control of body water level and electrolyte balance is extremely related to the hypothalamus function. When 0.5% or more of the body water is lost, we feel thirsty. The kidney can excrete about 20–28 L of water a day to control water load, and an excessive intake of water rarely causes hyponatremia [14], which provided with a significant renal function. A decrease in sodium level inhibits an antidiuretic hormone (ADH) secretion, and subsequently, the excreted amount of water via the kidney increases. In case of excessive water intake, hyponatremia develops only when the water intake amount exceeds the water excretion capacity of the kidney [15]. Many publications showed that hyponatremia is associated with prolonged hospitalization and severity, as well as mortality in a number of infectious diseases, especially COVID-19-infected patients [16, 17]. Yousaf et al. mentioned the mechanism of hyponatremia in COVID-19-infected patients secondary to SIADH and its pathophysiology. The hypothesis referring to increased interleukin-6 (IL-6) levels stimulates ADH release [18]. In Yousaf et al. case series, all three patients recovered with fluid restriction. However, it is essential to consider other possible etiologies as a cause of hyponatremia in COVID-19 patients. Hypovolemic hyponatremia should be distinguished from SIADH as these conditions employ different management guidelines and strategies; therefore, early diagnosis and management of hypovolemic hyponatremia affect morbidity and mortality [2]. In our presented case series study, hyponatremia is due to a dilutional one, and due to water intoxication, the diagnosis was done after exclusion of SIADH as well as taking a thorough history from the patient regarding excessive water intake; therefore, hyponatremia was corrected by fluid restriction. Anosmia-hyposmia and dysgeusia are common symptoms of mild-to-moderate COVID-19 cases. They are usually but not always reversible; it can be manifested in different and distorted taste feelings, and in our patient, dysgeusia felt as a bitterness of the tongue; therefore, the patients should try to overcome this sensation by frequent water drinking. We appreciate that the remaining three patients with excessive water intoxication thought that excessive water intake is beneficial to COVID-19 infection. We regard our presented cases were extremely rare, because our search for causes of hyponatremia due to excessive water intake was not existed in COVID-19 patients.

## Conclusions

The pathophysiological mechanisms of hyponatremia among COVID-19 patients are multifactorial, and it is not only secondary to SIADH but can also be due to other etiologies. Hyponatremia can be induced by excessive water drinking and considered an extremely rare reported cases.

## Data Availability

The data that support the findings of this study are available upon reasonable request from the corresponding author, A. Al-juboori. The data are not publicly available due to privacy restrictions.

## Abbreviations

COVID-19: Coronavirus disease of 2019
SIADH: Syndrome of inappropriate antidiuretic hormone
SARS-CoV-2: Severe acute respiratory syndrome coronavirus 2
ADH: Antidiuretic hormone
